# Reconstructive Orthopedic Surgery Using the Free Anterolateral Thigh Flap: Perspectives and Experience from a Single Trauma Center

**DOI:** 10.3390/life15121857

**Published:** 2025-12-03

**Authors:** Mariagrazia Cerrone, Virginia Cinelli, Chiara Comisi, Antonio Mascio, Federico Moretti, Camillo Fulchignoni, Elisabetta Pataia, Tommaso Greco, Giulio Maccauro, Carlo Perisano

**Affiliations:** 1 Department of Orthopedics and Geriatric Sciences, Catholic University of the Sacred Heart, Largo Francesco Vito 8, 00168 Rome, Italy; mariagraziacerrone13@gmail.com (M.C.); virginiacinelli23@gmail.com (V.C.); antonio.mascio87@gmail.com (A.M.); federicomoretti2020@gmail.com (F.M.); camillo.fulchignoni@gmail.com (C.F.); elisabetta.pataia@policlinicogemelli.it (E.P.); greco.tommaso@outlook.it (T.G.); giulio.maccauro@policlinicogemelli.it (G.M.); carlo.perisano@policlinicogemelli.it (C.P.); 2 Department of Orthopedics, Ageing and Rheumatological Sciences, Fondazione Policlinico Universitario A. Gemelli IRCCS, Largo Agostino Gemelli 8, 00168 Rome, Italy; 3 Department of Life Sciences, Health, and Healthcare Professions, Link Campus University, 00165 Rome, Italy

**Keywords:** anterolateral thigh flap, reconstructive surgical procedures, free tissue flaps, soft tissue injuries, orthopedic procedures

## Abstract

**Background**: Recent advances in microsurgical techniques have established free flaps as a cornerstone in complex orthopedic reconstructions, particularly in trauma, infection, and tumor resection cases requiring reliable soft tissue coverage for healing and functional recovery. Among them, the anterolateral thigh (ALT) flap is highly recognized for its versatility and consistency; **Methods**: We conducted a retrospective case series of five patients who underwent orthopedic reconstructive surgery using a free ALT flap in our department. Demographic, surgical, and clinical data were collected, and outcomes were evaluated and compared with the current literature. **Results**: The ALT flap proved to be a reliable option across a wide range of orthopedic conditions, providing well-vascularized and adaptable tissue for complex reconstructions. Clinical outcomes in our series were consistent with the favorable results reported in the literature. **Conclusions**: The ALT flap served as one of the most effective and versatile solutions for orthopedic reconstruction. Its adaptability to diverse clinical scenarios, combined with reproducible positive outcomes, supports its role as a preferred option for managing challenging soft tissue in orthopedics, ultimately contributing to improved function and recovery.

## 1. Introduction

Thanks to recent advances in microsurgical techniques, the use of free flaps has become a cornerstone in complex orthopedic reconstructions, especially in cases involving trauma, infections, or tumor resections, where soft tissue coverage is crucial for healing and functional recovery.

Among the most versatile and widely used options, the anterolateral thigh (ALT) flap stands out for its reliability and broad clinical applications. Originally described by Song et al. in 1984 [1], this flap is based on septocutaneous or musculocutaneous perforators from the lateral femoral circumflex system, with a consistently reliable vascular supply.

Several studies, such as those by Koshima [2], Kimura [3] and Wei [4], have highlighted the flexibility of the ALT flap, which can be harvested with or without fascia and adipose tissue, offering a wide range of tissue thicknesses and skin paddle sizes. Its vascular pedicle typically ranges from 12 to 16 cm in length, with arteries measuring about 2–3 mm in diameter, providing ideal conditions for microvascular anastomosis and resulting in high success rates; failure rates are often reported to be below 1% in the literature [3]. Additionally, the ALT flap can be designed as a sensate flap or as a flow-through flap, with minimal donor-site morbidity, often causing limited sensory loss or slight restrictions in thigh or knee movement [5].

One of the main challenges associated with the ALT flap is its tendency to be bulky, particularly in obese patients or women, where excess subcutaneous fat can complicate inset and potentially impair perfusion. Excessive volume can negatively affect both the esthetic outcome and the functional result, especially in reconstructing limbs or other areas requiring precise contouring. To overcome this, secondary procedures such as defatting or liposuction are often employed to improve both appearance and blood flow [6].

Ultimately, achieving the right balance between flap contour, vascular safety, and functional preservation is crucial for successful reconstruction [7]; therefore, a dedicated multidisciplinary team with advanced microsurgical expertise is essential to optimize outcomes and minimize complications.

The ALT flap is primarily used in reconstructive surgery, particularly for the treatment of large soft tissue defects following oncologic surgery [8], trauma, and complex injuries. It is extensively employed in reconstructing large areas such as the head and neck [9], including intraoral, mandibular–maxillary, tongue, and facial defects, periorbital region, hands, and cervical region. Its high versatility and reliability make it a preferred option in plastic and maxillofacial surgery for complex reconstructions; it is also gaining popularity in abdominal and pelvic reconstruction. In addition, it can be used as a pedicled flap for phallus or perineal reconstruction and has been increasingly employed in breast surgery as well [10].

The application of ALT free flap in orthopedics has been steadily expanding, driven by its numerous advantages that make it suitable for most orthopedic patients requiring extensive soft tissue reconstruction. This trend has also been supported by the evolution of orthoplastic surgery, which has integrated surgeons trained in reconstructive soft tissue surgery and microsurgery into multidisciplinary teams within specialized referral centers [11].

In this series of clinical cases, we present our experience in the field of orthopedics as a specialized reference center with a dedicated orthoplastic team, highlighting the versatility of the ALT flap and the positive clinical outcomes achieved. Our goal is to contribute to the existing body of knowledge by providing practical insights into the application of this flap, based on our patient cohort and the implications derived from our work.

## 2. Materials and Methods

### 2.1. Study Design

We conducted an observational, retrospective single-center cases series according to the STrengthening the Preferred Reporting Of Case Series in Surgery (PROCESS) 2023 guidelines [12]. We included all consecutive patients undergoing orthopedic reconstruction with a free ALT flap in our department, a second-level referral trauma and oncological center. Data were analyzed in a period extended from January 2023 to February 2025, ensuring that all patients had at least ten months of follow-up. In line with institutional protocols, all patients provided informed consent for surgery and for the collection of clinical data for scientific purposes upon admission and before the procedure. All patients were administered an evaluation questionnaire in which they were asked to provide a subjective assessment (poor, fair, good, or excellent) of their functional recovery and esthetic outcome at 6 months after surgery. In addition, at the 6-month outpatient follow-up, the examining surgeon, who was the same expert surgeon and participated both in the operative procedures and in the follow-up checks to maximize the validity of our results, provided an independent subjective assessment (poor, fair, good, or excellent) of the patient’s function and esthetic.

### 2.2. Inclusion and Exclusion Criteria

We included (i) patients of all ages, (ii) who underwent a reconstructive procedure with a free ALT flap, (iii) with different diagnosis leading to wide soft tissues loss (complex trauma, oncologic resections, or osteomyelitis). They were selected to highlight the remarkable versatility of the free ALT flap in orthopedic practice. Exclusion criteria were as follows: (i) amputation within 6 months from the reconstruction surgery; (ii) incomplete data or loss to follow-up.

### 2.3. Data Collection and Patients Setting

Relevant clinical, demographic, and perioperative data were retrospectively collected from the institutional electronic medical records and operative reports of all patients meeting the inclusion criteria. Data sources included hospital discharge summaries, surgical charts, pathology reports, and outpatient follow-up documentation.

Diagnostic imaging data were reviewed to assess the underlying pathology and the soft tissue involvement, including X-ray, Computed Tomography (CT) scan, computed tomography angiography (CTA) and, when available, Magnetic Resonance Imaging (MRI).

Surgery was performed by the same expert surgeons and their specialized team. ALT flap was always harvested, identifying and dissecting perforator vessels from the descending branch of the lateral femoral circumflex artery. The flap was raised in a free fashion as a fasciocutaneous or myocutaneous flap according to the patient’s need for the reconstruction of the soft tissue defect. The process required, at all times, careful preoperative planning and intraoperative dissection to ensure the vascular supply is sufficient for the size of the flap, with the donor site then closed by a first-intention suture or a skin graft for larger flaps. The preoperative planning included a CT angiography to perform a detailed evaluation of the vascular anatomy and to identify the most suitable perforator branch. Intraoperatively, the use of a sterile Doppler probe confirmed the accuracy of the preoperative planning.

### 2.4. Outcome and Statistical Analysis

The primary outcome of this study was flap survival. Secondary outcomes included postoperative complications, donor-site morbidity, as well as functional recovery and esthetic results.

Descriptive statistics were used: continuous variables were reported as mean (range), and categorical variables as absolute numbers and percentages. Data were analyzed using Microsoft Excel (2021 18.0) and SPSS (IBM Corp., Armonk, NY, USA).

## 3. Results

A total of six patients who met all the inclusion criteria, comprising five males and one female, underwent reconstructive surgery of this soft tissue defect during our study period. Underlying clinical conditions varied: one patient presented with post-burn sequelae, one with a complex open fracture, three required reconstruction following oncologic resection, and one was treated for the consequences of tibial chronic osteomyelitis. Reconstruction involved the upper limb in four cases and the lower limb in two. The mean patient age was 42.8 years ranging from 7 to 72 years.

Comorbidities potentially affecting outcomes were assessed, especially including diabetes (affecting none of the patients), obesity (in 16.7%), and smoking (in 20%). Demographic and clinical data are summarized in Table 1.

No significant differences were found between patients with comorbidities and those without in terms of clinical and esthetic outcomes.

Among the six cases analyzed, complete flap survival was achieved in three patients (50%), while partial flap necrosis occurred in the remaining three (50%). No cases of flap failure, surgical site infection, or donor-site morbidity were observed. Wound dehiscence, retractile scars, and the need for reoperation were each reported in one patient (16.67%) (Table 2).

Instead, Table 3 presents the results of the functional recovery and esthetic outcome assessment following free ALT flap reconstruction, as evaluated by both the patients and the operating surgeon at the 6-month outpatient follow-up.

Each patient and the surgeon were asked to rate functional recovery as poor, fair, good, or excellent, using the same scale for esthetic outcome, including both the recipient and donor sites.

Regarding functional recovery, patient and surgeon ratings were concordant in four cases; in one case, the patient’s rating was higher than the surgeon’s, and in another, the opposite occurred.

For the esthetic outcome, ratings were identical in half of the cases; while in the remaining half, patients rated the result higher than the surgeon. In no case did the surgeon’s esthetic evaluation exceed that of the patient.

## 4. Cases

### 4.1. Case 1: Retracting Scars Following an Explosion Injury in a Pediatric Patient

A 7-year-old boy was referred to our emergency department by the Libyan embassy following a domestic gas cylinder explosion, which had caused third-degree burns over approximately 55% of the total body surface about one month earlier. The patient underwent several fasciotomies due to compartment syndrome and experienced two episodes of central venous catheter-related sepsis, which complicated his management.

Approximately six months after the injury, once his systemic condition had stabilized, the Pediatric Department requested a consultation from our Hand Surgery Unit. At presentation, he exhibited severe retractile scarring, elbows fixed at 90° flexion with limited active extension, wrists held in extension with fixed flexion of the index finger and ineffective pinch, and partial reducibility of flexion at the metacarpophalangeal and proximal interphalangeal joints.

To improve upper limb function, limited by the retractile scars, we decided to operate on the right hand first (the dominant hand), with future surgery planned for the left. The procedure involved excision of the skin and subcutaneous scar tissue encasing the extensor apparatus of the right hand, followed by tenolysis. Complete passive extension and approximately 20° of flexion were achieved intraoperatively. However, in neutral wrist position, a 5 × 9 cm skin defect remained, which was reconstructed with a free fasciocutaneous ALT flap (based on one perforator vessel) anastomosed to the radial artery, which was intact and presented an adequate caliber. A temporary radiocarpal arthrodesis with K wires was performed to maintain the achieved flexion and minimize the risk of recurrent contracture. Primary wound closure was performed at the donor site.

**Figure 1:**
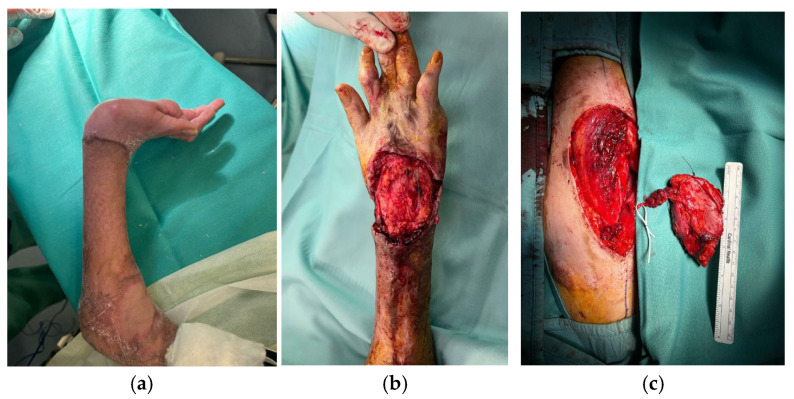
7-year-old patient with retracting scars on the right hand and wrist (**a**). Excision of the scars, tenolysis, and preparation of the recipient site (**b**). Harvesting of the ALT flap from the left thigh (**c**).

**Figure 2:**
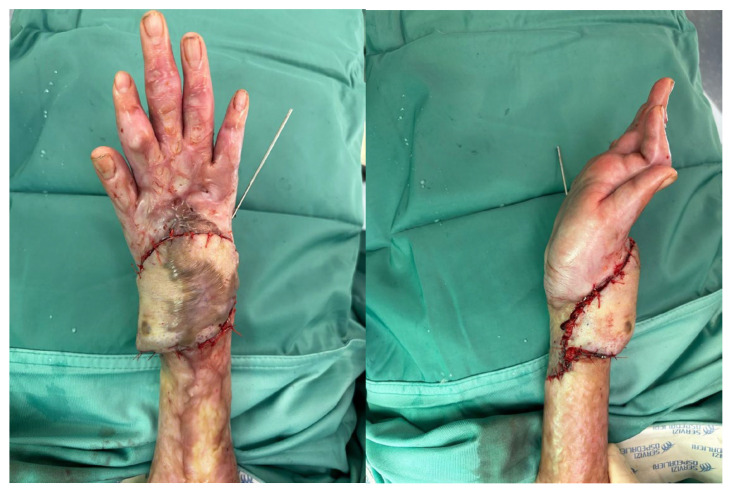
ALT flap for reconstruction of a soft tissue defect of the wrist; intraoperative images.

**Figure 3:**
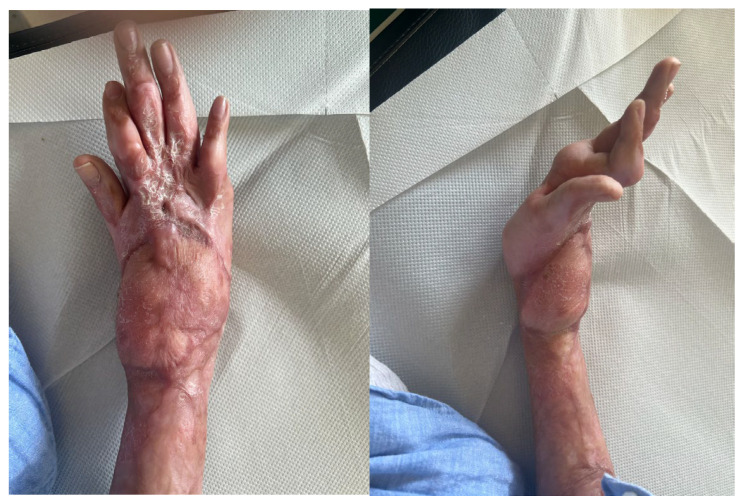
ALT flap for reconstruction of a soft tissue defect of the wrist, 30-day follow-up.

Thirty days later, following Kirschner wire removal, the flap was completely healed with no complications, and the child began motor rehabilitation in the Rehabilitation Unit to optimize wrist and finger mobility (Figure 1, Figure 2 and Figure 3).

Follow-up evaluations were performed at 3, 6, and 10 months. The flap survived completely without any signs of necrosis or local infection. After physiotherapy, the patient achieved a good functional outcome, with satisfactory recovery of wrist flexion and extension.

### 4.2. Case 2: Complex Open Fracture of the Elbow

A 31-year-old man presented to our emergency department with a right elbow gunshot wound sustained accidentally while hunting. Past medical history was unremarkable. He reported a Gustilo IIIB open comminuted fracture of the right elbow with a large dorsal-ulnar laceration and articular instability. Distal pulses were present; active wrist and finger flexion/extension were preserved, with a deficit in finger ab/adduction; sensation was intact except for paresthesia of the fifth finger. Upper limb CT angiography showed all arterial branches were patent.

Emergency surgery included debridement of soft tissues and external fixation of the elbow fracture. After 72 h, he underwent a second-look procedure with intraoperative biopsies, which were positive for Enterobacter cloacae; first-intention wound closure was performed, and targeted antibiotic therapy was initiated.

At a follow-up visit approximately 20 days later, wound dehiscence occurred with extensive bone exposure. To ensure proper coverage of the bone and closure of the skin, a pedicled radial forearm flap was performed.

**Figure 4:**
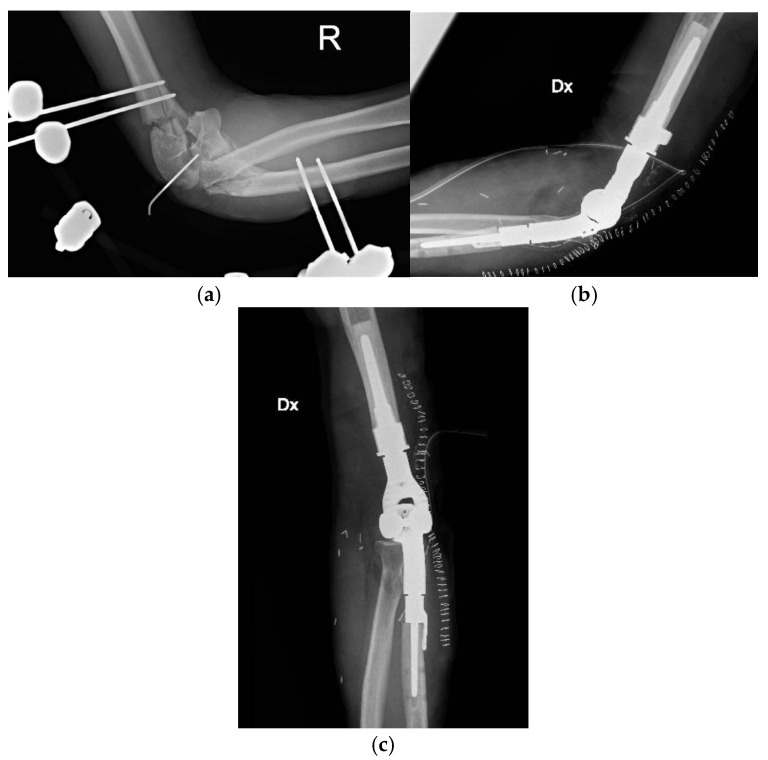
Complex open fracture of the right elbow (**a**). Emergency stabilization with external fixator (**b**). Definitive reconstruction with custom-made elbow prosthesis (**c**).

**Figure 5:**
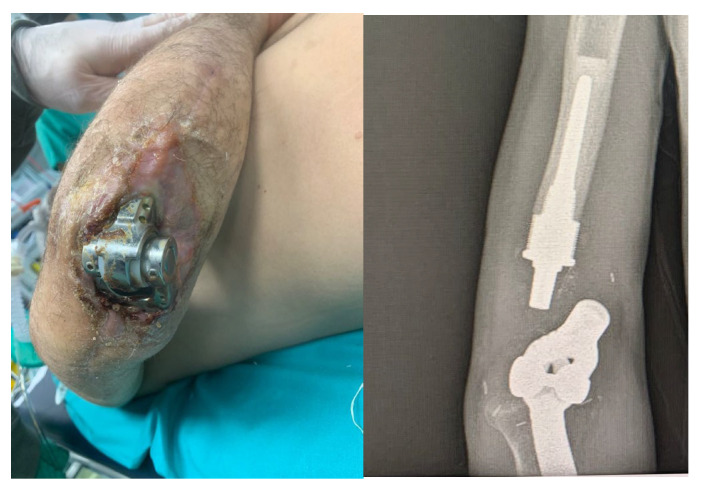
Wound dehiscence with prosthesis exposure and disassembly of the humeral prosthetic components at about 9 months from the injury.

About eight months after the injury, with the infection resolved and local skin healed, definitive treatment with a custom-made Pantheon elbow resection prosthesis was undertaken (Figure 4). Thirty days later, wound dehiscence recurred with prosthesis exposure, and X-rays revealed disassembly of the humeral prosthetic components (Figure 5). Revision surgery was performed, and soft tissue coverage was achieved with a free fascial-myocutaneous ALT flap harvested from the left thigh based on one perforator vessel anastomosed to a branch of the ulnar recurrent artery and its comitant veins. Perfusion test results were positive. The donor site was closed by primary intention.

**Figure 6:**
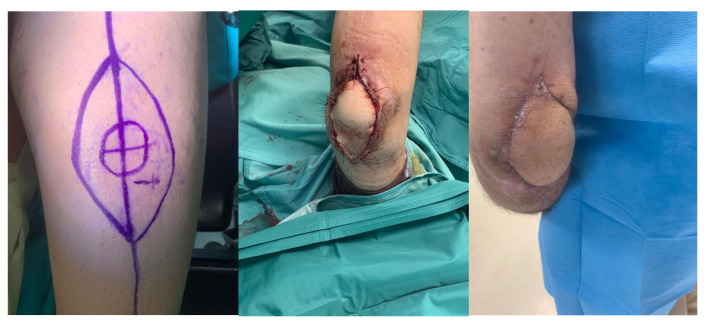
Revision surgery performed and soft tissue coverage of the elbow achieved with a free fascial-myocutaneous ALT flap.

At one-month follow-up, the patient presented with limited elbow range of motion (20° deficit of flexion and 50° deficit of extension) and two small eschars of the distal portion of the flap, treated with advanced dressings; at three months following appointment at the clinic, the eschars had resolved, but ROM remained severely restricted, with the patient reporting non-adherence to prescribed physiotherapy (Figure 6).

At 6-month follow-up, the flap was completely healed, and the joint range of motion had improved.

### 4.3. Case 3: Solitary Fibrous Tumor of the Elbow

A 62-year-old man was referred to our unit for a left elbow mass, partially excised elsewhere, with histology showing a high-risk solitary fibrous tumor. Radical excision was planned, and due to the defect size (12 × 5.8 × 5.8 cm), soft tissue reconstruction with an ALT flap was scheduled. Intraoperative frozen section was executed over the bioptical samples collected from the resection margins testing negative for tumor cell presence. We were not able to anastomose to veins at the recipient site due to the absence of adequate vessels, so two Penrose drainages were implemented to improve the venous drainage. Vascularization and refill time of the flap appeared satisfactory at the end of the procedure. First-intention suture of the donor site was performed. The left arm was immobilized in flexion position by a plaster splint.

At 15-day follow-up, the flap was viable with minor superficial skin necrosis, which was treated with a skin graft and healed uneventfully. The splint was removed three weeks after surgery, when soft tissues were stable and the graft was almost healed. The patient started a rehabilitation program aimed at regaining full elbow mobility.

**Figure 7:**
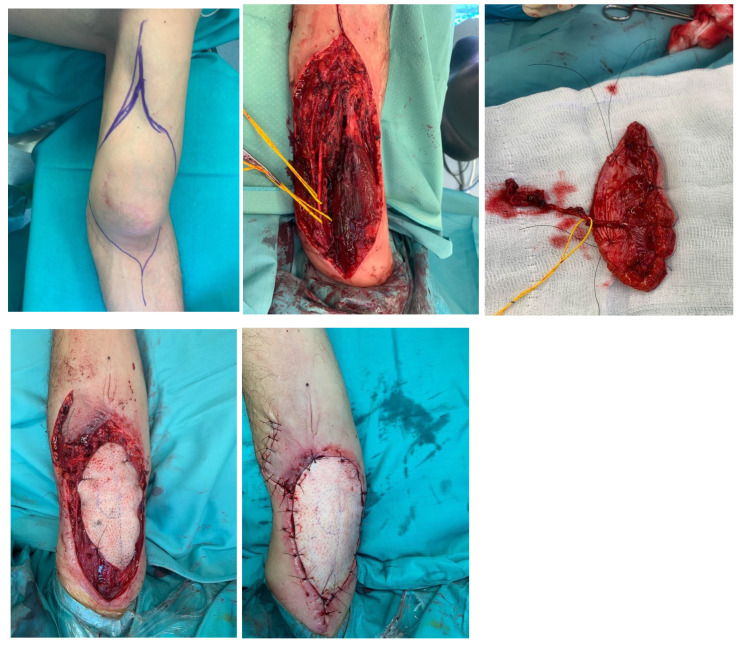
Soft tissue loss of the elbow. Reconstruction with an ALT flap after resection of a solitary fibrous tumor.

Four months later, the patient developed a retractile scar-limiting elbow motion; scar excision with skin graft reconstruction was performed. Intraoperative biopsies performed were negative for tumor recurrence. The patient resumed rehabilitation, achieving full elbow ROM at the six-month follow-up appointment (Figure 7).

## 5. Discussion

The free ALT flap is a widely used flap in reconstructive surgery: it is utilized for the reconstruction of extensive defects of the lower abdomen, inguinal region, and head and neck area [14]. It has also been introduced in the orthopedic field for the reconstruction of large defects of the upper and lower limbs [15], where local flaps proved to be insufficient. Free flaps represent the most suitable option when local conditions, such as complex trauma with compromised local vascularization, extensive soft tissue loss, local infection, previous local flap failure, or absence of local options, do not allow harvesting of a viable local flap to cover the defect.

The examples reported in this case series demonstrate the extensive versatility of the ALT flap and the favorable outcomes associated with its use. Although these cases are not intended to represent novelty, they illustrate practical considerations that are rarely detailed in the literature and may assist surgeons facing similar reconstructive challenges.

Our aim is to present the authors’ perspective and accumulated experience with the use of the free ALT flap in reconstructive orthopedic surgery.

In the first reported case, the ALT flap was employed for retractive scars treatment in a 7-year-old child following a burn injury. In the same context, the study by Lee et al. [16] described seven cases of foot scar contractures with severe toe deformities caused by burns. Patients underwent surgeries such as tendon lengthening, capsule release, and bone plating, along with reconstruction using an ALT flap. All flaps healed without complications, and patients could walk pain-free post-surgery. Around 71% reported high esthetic satisfaction. Similarly, in our case, the esthetic result was satisfactory and the functional outcome acceptable, confirming the effectiveness of the ALT flap in treating retractive scars, even in children.

ALT use in children is rare because of small blood vessels and rapid physiological changes. It has been shown to have a higher risk of donor site hyperplasia and the need for additional procedures. The study by Shi et al. [17] explores its effectiveness in pediatric extremity reconstruction. It included 26 children with limb defects involving exposed bones or tendons from injury or cicatricial conditions. Postoperative complications included vascular crisis, flap necrosis, infection, pressure ulcer, and dehiscence. Eleven patients (42.3%) had scar hyperplasia at the donor site; 34 reoperations addressed various issues. Up to the last follow-up, none of these postoperative complications were observed in our patient, and healing progressed uneventfully.

In three cases, the ALT flap was chosen for reconstructive purposes in oncologic patients. One of these cases involves a patient with a solitary fibrous tumor of the elbow. It is a rare tumor and in an unusual location. Mach et al. [18] report a similar case, though reconstructive flaps were not required. However, given the high vascularization of the mass, local options would likely have been unfeasible: vessel ligation or embolization could have compromised pedicle viability, making a free flap such as an ALT the most appropriate option if soft tissue reconstruction had been needed.

Historically, limb amputation was frequently the treatment of choice for tumor-related lesions; however, thanks to advances in chemoradiotherapy and surgical techniques, there is now a growing preference for limb-sparing approaches. Nonetheless, achieving a radical tumor excision often requires extensive tissue removal, and the resulting soft tissue defects may not always be adequately reconstructed with local tissues alone. Therefore, free flaps, such as the ALT, are essential for salvage surgeries in these patients. In a case series by Qiao et al. [19], the ALT flap was successfully used in the treatment of 11 cases of soft tissue sarcoma of the lower limbs, with satisfactory results in 10 cases.

The potential applications of the ALT flap in oncologic surgery extend beyond simple soft tissue coverage [20], as the flap can also be used for more complex reconstructions, such as simultaneous vascular reconstruction and repair of soft tissue defects following oncologic resection.

Miyamoto et al. successfully used a free ALT flap to reconstruct the popliteal artery and a soft tissue loss, simultaneously, for limb salvage in two cases of knee sarcomas [21]. Reconstruction of the popliteal artery and soft tissue coverage should be performed simultaneously for limb salvage. They used a free flow-through ALT flap as a bypass flap: the popliteal artery and vein were reconstructed using the branches of the lateral circumflex femoral arterial system, restoring leg vascularization and preserving the limb.

With the aim of highlighting the great versatility of the free ALT flap in the orthopedic field, we can refer to the study conducted by When et al. It demonstrated that combining ALT free flaps with kickstand external fixation enhances diabetic foot ulcer management, accelerating healing, improving 6-month functional outcomes, and achieving a 91.7% flap success rate. This orthoplastic approach is a safe and effective limb salvage strategy that supports faster recovery [22].

Atilgan et al. also demonstrated excellent outcomes and significant versatility of the ALT flap, which was used for reconstructing soft tissue defects of the lower extremities. The most common etiologies included traffic accidents, gunshot wounds, electrical burns, open fractures, infections, diabetic foot, and skin tumors. Out of 23 patients, there was only one flap failure, underscoring the high success rate and reliability of the ALT flap in this setting [23].

Several free flaps can be effectively used in orthoplastic reconstruction, with the choice guided by lesion characteristics, defect size, and patient condition. The gluteus maximus flap is advantageous in hip surgery, as it preserves origin, insertion, and innervation, minimizing functional loss, though it may compromise joint stability [24]. The rectus abdominis flap maintains lower limb strength but may be less suitable in elderly patients due to muscle atrophy or fibrosis [25]. For extensive dead spaces, the free latissimus dorsi myocutaneous flap provides ample tissue volume and remains a preferred option, especially after failure of local or regional flaps, despite its greater technical complexity and operative time [26,27]. Moreover, a surgeon’s experience and preference also play an important role in choosing the covering strategy and achieving the best possible outcome for the patient.

Demirtas [28] and Philandrianos [29] demonstrated that the free ALT flap offers better esthetic results at the recipient site compared to other flaps such as the latissimus dorsi flap or muscle flaps, although the other options also provide good functional outcomes.

Several modifications to the original ALT flap design have been described in the literature, aiming to optimize both functional and esthetic outcomes. In particular, achieving primary closure of the donor site, avoiding split-thickness skin grafting, has been emphasized as a key factor for improved results [5]. When a longer pedicle is required, the flap can be designed with an eccentrically positioned perforator located distally, allowing greater flexibility in positioning [30]. Additionally, when two perforators arise from the main pedicle, the ALT flap can be split into two subunits, maximizing tissue usage while further minimizing donor-site morbidity [31]. However, it is important to note that in a small percentage of patients (up to 5.4%), ALT perforators may be absent, which poses a potential limitation [7,32]. Recently, new technical variants have been proposed, such as the lazy-S incision, which provides better exposure during flap harvesting, greater flexibility in customizing the skin paddle based on the best available perforator, and reduced tension during donor site closure. Another innovation is the teardrop-shaped ALT flap [33], which offers better adaptation to complex defects with more balanced tissue distribution. The presence of a tail in the teardrop-shaped ALT flap facilitates contouring of the flap on the lower leg, particularly in small- to medium-sized defects located near the joints, where the potential for contour adjustment using surrounding tissues is limited. By harvesting a flap slightly longer than the defect, the “tail” can be positioned over the anastomotic site, thereby protecting the pedicle from pressure due to primary closure and postoperative edema of adjacent tissues, while also improving the overall contour.

Among other considerations, Bota et al. highlight that the ALT flap can be used to cover very large defects, significantly larger than those reported in our case series, when harvested in its extended form [34]. This further underscores the remarkable versatility of the ALT flap, which, thanks to its reliable perforators and the possibility of tailoring both shape and size, makes it suitable for a broad spectrum of lower-limb defects, including high-energy injuries with extensive soft tissue loss.

One limitation of ALT flaps in large reconstructions is the absence of a bone component, which restricts its use when osseous restoration is required. In such cases, vascularized bone flaps—such as the fibular flap—may offer greater effectiveness. Although extended ALT flap variants incorporating iliac bone have been described [10], further research is needed to validate their reliability and clinical applications.

Another aspect of the versatility of the ALT flap lies in its ability to be harvested as a composite flap, such as including a vascularized fascia lata component, as described by Lee et al. in the treatment of an infected Achilles tendinitis [35]. In their case report, the free ALT flap offered several advantages, including easy healing, high resistance to infection, low donor-site morbidity, and high cost-effectiveness due to the possibility of achieving definitive reconstruction in a single procedure. Additional benefits included reduced scarring and adhesion, as well as improved tendon gliding and excursion.

The most common complications reported include venous congestion, infection, and partial flap necrosis. With the aim of detecting postoperative complications early and minimizing their impact on flap viability, all patients undergo serial (weekly) outpatient follow-up visits. Although these rarely threaten flap survival, they may require further surgical management.

Ikeguchi et al. reported venous anastomosis revision for the management of venous congestion and debridement with irrigation for an MRSA infection, with both complications observed in their series of 17 patients who underwent ALT flap reconstruction for upper-limb soft tissue defects [36].

In our cases, we encountered three instances of partial flap-edge necrosis, successfully managed without compromising flap viability through a surgical debridement followed by skin grafting in one patient or, in the other cases, with advanced outpatient dressings.

There is currently no internationally accepted tool for evaluating the esthetic outcome after flap reconstruction.

Regarding the assessment of esthetic and functional results, the presence of multiple evaluation scales in the literature, the variability in defect location (often near joints), and the inclusion of subjective items based on the surgeon’s or patient’s judgment make comparison between different patient series particularly challenging.

Ellabban et al. investigated the outcomes of complex upper limb wound reconstructions using thinned ALT flaps [37]. They evaluated the esthetic and functional results in 18 patients using a 5-point Likert scale, the QuickDASH score, passive range of motion, and power grip at 12 months, postoperatively obtaining good results in all patients.

Considering the points discussed above, it is evident that comparing our functional and esthetic outcomes with those reported in the literature remains challenging. Although our evaluations are based on subjective assessments, we believe our findings remain meaningful because they allow us to explore key aspects of patient recovery. By directly involving patients in the evaluation process, we can better understand their level of satisfaction with the final outcome. Our goal was to determine how satisfied patients were with their return to daily activities.

Nevertheless, generally positive results, despite some difficulties during the healing process, were also observed in our series, with 92% of patients rating their functional or esthetic recovery as *good* or *excellent*, and 75% of surgeons providing the same evaluation.

This case series highlights the reliability of the ALT flap for complex orthopedic reconstructions across different etiologies. At the time of the latest follow-up, all patients were regularly monitored and in good clinical condition, with stable soft tissue coverage, full survival flap, and no evidence of complications. All individuals were able to resume their daily and occupational activities, achieving satisfactory functional recovery and esthetic results.

Despite the small sample size, the consistent outcomes confirm its versatility and low donor-site morbidity. Larger multicenter prospective studies are useful to define standardized protocols for flap selection and outcome assessment in orthoplastic reconstruction. Finally, collaborative research combining biomechanical modeling, machine learning-based outcome prediction, and long-term functional follow-up could provide deeper insight into factors influencing flap survival, complication rates, and overall rehabilitation outcomes. Furthermore, multidisciplinary efforts will be crucial to consolidate evidence-based guidelines and validate the best reconstructive strategies for patients with complex orthopedic soft tissue defects.

## 6. Conclusions

In conclusion, the ALT flap stands out as one of the most effective and reliable options in orthopedic surgery, thanks to its ability to provide highly vascularized and versatile tissue. Its adaptability to various reconstructive needs, combined with proven positive clinical outcomes, makes it a preferred choice for managing complex soft tissue defects. The available data, including those from our case series, demonstrate that the ALT flap can significantly contribute to improving functional outcomes and promoting rapid, effective recovery in patients with challenging orthopedic injuries.

## Figures and Tables

**Table 1:** Demographic and clinical characteristics of the study population.

Patient	Cause	Site	Sex	Age	Comorbidity (Excluding Diabetes)	Diabetes	BMI > 25 (kg/m^2^)	Smoke
**1**	Post-burn sequelae	Arm	M	7	No	No	No	No
**2**	Open fracture	Elbow	M	32	No	No	No	Yes
**3**	Oncologic	Elbow	M	62	Yes	No	No	No
**4**	Oncologic	Forearm	F	72	Yes	No	No	No
**5**	Oncologic	Hindfoot [13]	M	54	No	No	Yes	No
**6**	Consequences of osteomyelitis	Leg	M	30	No	No	No	Yes

**Table 2:** Summary of free ALT flap outcomes, including the primary outcome (flap survival) and selected secondary outcomes such as postoperative complications (we analyzed the main postoperative complications reported in the literature following free ALT flap reconstruction) and donor-site morbidity.

Outcome	Number (N)	Percentage (%)
Flap Failure	0	0%
Complete Flap Survival	3	50%
Partial Flap Necrosis	3	50%
Surgical Site Infection	0	0%
Wound Dehiscence	1	16.67%
Retractile scars	1	16.67%
Reoperation Rate	1	16.67%
Donor-Site Morbidity	0	0%

**Table 3:** Patient- and surgeon-reported functional and esthetic outcomes at 6 months after free ALT flap surgery.

Patient	Patient’s Functional Assessment	Patient’s Esthetic Assessment	Surgeon’s Functional Assessment	Surgeon’s Esthetic Assessment
**1**	Fair	Good	Fair	Fair
**2**	Good	Excellent	Fair	Excellent
**3**	Excellent	Excellent	Excellent	Good
**4**	Good	Good	Good	Good
**5**	Good	Excellent	Excellent	Good
**6**	Good	Good	Good	Good

## Data Availability

The original contributions presented in this study are included in the article. Further inquiries can be directed to the corresponding author.

## Abbreviations

The following abbreviations are used in this manuscript:

ALT	Anterolateral thigh
CT	Computed Tomography
CTA	Computed Tomography Angiography
MRI	Magnetic Resonance Imaging
MRSA	Methicillin-resistant *Staphylococcus aureus*
ROM	Range of motion

## References

[1] Song Y., Chen G., Song Y. (1984). The free thigh flap: A new free flap concept based on the septocutaneous artery. Br. J. Plast. Surg..

[2] Koshima I., Kawada S., Etoh H., Kawamura S., Moriguchi T., Sonoh H. (1995). Flow-through anterior thigh flaps for one-stage reconstruction of soft-tissue defects and revascularization of ischemic extremities. Plast. Reconstr. Surg..

[3] Kimura N., Satoh K., Hasumi T., Ostuka T. (2001). Clinical application of the free thin anterolateral thigh flap in 31 consecutive patients. Plast. Reconstr. Surg..

[4] Wei F., Jain V., Celik N., Chen H., Chuang D.C.-C., Lin C. (2002). Have we found an ideal soft-tissue flap? An experience with 672 anterolateral thigh flaps. Plast. Reconstr. Surg..

[5] Kimata Y., Uchiyama K., Ebihara S., Sakuraba M., Iida H., Nakatsuka T., Harii K. (2000). Anterolateral thigh flap donor-site complications and morbidity. Plast. Reconstr. Surg..

[6] Ross G., Dunn R., Kirkpatrick J., Koshy C., Alkureishi L., Bennett N., Soutar D., Camilleri I. (2003). To thin or not to thin: The use of the anterolateral thigh flap in the reconstruction of intraoral defects. Br. J. Plast. Surg..

[7] Kimata Y., Uchiyama K., Ebihara S., Nakatsuka T., Harii K. (1998). Anatomic variations and technical problems of the anterolateral thigh flap: A report of 74 cases. Plast. Reconstr. Surg..

[8] Saracco M., Cerrone M.G., Vavalle G., Vitiello R., El Ezzo O., Maccauro G., Pataia E. (2025). Orthoplastic approach to limb salvage surgery in oncology: Types of flaps and surgical timing. Folia Med..

[9] Smith R.K., Wykes J., Martin D.T., Niles N. (2017). Perforator variability in the anterolateral thigh free flap: A systematic review. Surg. Radiol. Anat..

[10] Ali R.S., Bluebond-Langner R., Rodriguez E.D., Cheng M.-H. (2009). The Versatility of the Anterolateral Thigh Flap. Plast. Reconstr. Surg..

[11] Fulchignoni C., Pietramala S., Lopez I., Mazzella G.G., Comisi C., Perisano C., Rocchi L., Greco T. (2024). Surgical outcomes and complications of custom-made prostheses in upper limb oncological reconstruction: A systematic review. J. Funct. Morphol. Kinesiol..

[12] Mathew G., Sohrabi C., Franchi T., Nicola M., Kerwan A., Agha R., PROCESS Group (2023). Preferred Reporting Of Case Series in Surgery (PROCESS) 2023 guidelines. Int. J. Surg..

[13] Comisi C., Greco T., Fulchignoni C., Mascio A., Farine F., Troiano E., Mondanelli N., Menichini G., Pataia E., Maccauro G. (2025). A rare case of high-grade synovial sarcoma of the hindfoot. Med. Glas..

[14] Lin C., Wang C., Ou K., Chang S., Dai N., Chen S., Chen T., Tzeng Y. (2017). Clinical applications of the pedicled anterolateral thigh flap in reconstruction. ANZ J. Surg..

[15] Wong C.-H., Ong Y.S., Wei F.-C. (2012). The anterolateral thigh–vastus lateralis conjoint flap for complex defects of the lower limb. J. Plast. Reconstr. Aesthet. Surg..

[16] Lee S.H., An S.J., Kim N.R., Kim U.J., Kim J.I. (2016). Reconstruction of postburn contracture of the forefoot using the anterolateral thigh flap. Clin. Orthop..

[17] Shi Y., Xu Y., Zhu Y., Yang X., Wang T., Cui Y., Zhang X., He X. (2022). Microsurgical anterolateral thigh flap for reconstruction of extremity soft tissue defects in pediatric patients. Ann. Plast. Surg..

[18] Mach M., Ostrowski T., Maciejewski K., Ostrowski R., Gałązka Z. (2025). Solitary Fibrous Tumor of the Right Elbow: A Case Report. Cureus.

[19] Qiao J., Mao H., Wen L., Xu L., Zhu Z., Qiu Y., Xiong J., Wang S. (2022). Reconstruction of soft tissue defect with a free vascularized anterolateral thigh flap after resection of soft tissue sarcoma in extremities. Orthop. Surg..

[20] Fulchignoni C., Cianni L., Matrangolo M.R., Cerrone M., Cavola F., Pataia E., Vitiello R., Maccauro G., Farsetti P., Rovere G. (2024). A two-step approach to the surgical treatment of soft-tissue sarcomas. Curr. Oncol..

[21] Miyamoto S., Fujiki M., Nakatani F., Sakisaka M., Sakuraba M. (2015). Free flow-through anterolateral thigh flap for complex knee defect including the popliteal artery. Microsurgery.

[22] Wen J., Zhou Z., Boey J., Yu L., Marei A.E., Meng F., Xiao Y., Zeng H., Wan S. (2025). Clinical evaluation of orthoplastic limb salvage protocol using anterolateral femoral free flap and kickstand fixation: A retrospective case-control study. Foot.

[23] Atilgan N., Ipek B., Duman N., Orhan O., Yilmaz M. (2023). Can anterolateral thigh flap be a rescuer in lower extremity injuries?. Eur. Rev. Med. Pharmacol. Sci..

[24] Ricciardi B.F., Henderson P.W., McLawhorn A.S., Westrich G.H., Bostrom M.P., Gayle L.B. (2017). Gluteus maximus advancement flap procedure for reconstruction of posterior soft tissue deficiency in revision total hip arthroplasty. Orthopedics.

[25] Ross D.A., Gürlek A., Gheradini G., Miller M.J. (1998). Transosseous transposition of a pedicled rectus abdominis flap to cover hip wounds. Eur. J. Surg. Oncol. EJSO.

[26] Rovere G., De Mauro D., D’oRio M., Fulchignoni C., Matrangolo M.R., Perisano C., Ziranu A., Pataia E. (2021). Use of muscular flaps for the treatment of hip prosthetic joint infection: A systematic review. BMC Musculoskelet. Disord..

[27] Rovere G., Smakaj A., Calori S., Barbaliscia M., Ziranu A., Pataia E., Maccauro G., De Mauro D., Liuzza F. (2022). Use of muscular flaps for the treatment of knee prosthetic joint infection: A systematic review. Orthop. Rev..

[28] Demirtas Y., Kelahmetoglu O., Cifci M., Tayfur V., Demir A., Guneren E. (2010). Comparison of free anterolateral thigh flaps and free muscle-musculocutaneous flaps in soft tissue reconstruction of lower extremity. Microsurgery.

[29] Philandrianos C., Moullot P., Gay A.M., Bertrand B., Legré R., Kerfant N., Casanova D. (2018). Soft tissue coverage in distal lower extremity open fractures: Comparison of free anterolateral thigh and free latissimus dorsi flaps. J. Reconstr. Microsurg..

[30] Sofiadellis F., Liu D.S., Webb A., MacGill K., Rozen W.M., Ashton M.W. (2012). Fasciocutaneous free flaps are more reliable than muscle free flaps in lower limb trauma reconstruction: Experience in a single trauma center. J. Reconstr. Microsurg..

[31] Chang N.-J., Waughlock N., Kao D., Lin C.-H., Lin C.-H., Hsu C.-C. (2011). Efficient design of split anterolateral thigh flap in extremity reconstruction. Plast. Reconstr. Surg..

[32] Choi K., Cho J., Park M., Park D.H., Lee I.J. (2016). Knee and ankle reconstruction with reverse anterolateral thigh and free anterolateral thigh flap from one donor site. Int. J. Low. Extrem. Wounds.

[33] Razzano S., Ramadan S., Figus A., Haywood R.M. (2018). Tear drop-free anterolateral thigh flap, a versatile design for lower limb reconstruction after trauma. Microsurgery.

[34] Bota O., Meier F., Garzarolli M., Schaser K.-D., Dragu A., Taqatqeh F., Fritzsche H. (2023). Lower leg reconstruction after resection of a squamous cell carcinoma on necrobiosis lipoidica with a pedicled fibula and an extended anterolateral thigh flap—A case report. World J. Surg. Oncol..

[35] Lee Y.-K., Lee M. (2018). Treatment of infected Achilles tendinitis and overlying soft tissue defect using an anterolateral thigh free flap in an elderly patient: A case report. Medicine.

[36] Ikeguchi R., Noguchi T., Ando M., Yoshimoto K., Sakamoto D., Matsuda S. (2022). Anterolateral thigh flap for upper extremity reconstruction in older patients. Microsurgery.

[37] Ellabban M.A., Gomaa A.A., Moghazy A.M., Elbadawy M.A., Adly O.A. (2021). Aesthetic and functional outcomes of thinned anterolateral thigh flap in reconstruction of complex wounds of the upper limb. J. Hand Surg. Eur..

